# Oxidative and/or Inflammatory Thrust Induced by Silver Nanoparticles in Rabbits: Effect of Vitamin E or NSAID Administration on Semen Parameters

**DOI:** 10.1155/2020/6664062

**Published:** 2020-12-26

**Authors:** Collodel Giulia, Mattioli Simona, Moretti Elena, Cerretani Daniela, Micheli Lucia, Fiaschi Anna Ida, Menchetti Laura, Brecchia Gabriele, Castellini Cesare

**Affiliations:** ^1^Department of Molecular and Developmental Medicine, University of Siena, Policlinico Santa Maria alle Scotte, Viale Bracci, 14, 53100 Siena, Italy; ^2^Department of Agricultural, Environmental and Food Science, University of Perugia, Borgo XX Giugno, 74, 06123 Perugia, Italy; ^3^Department of Medical and Surgical Sciences and Neurosciences, University of Siena, Policlinico Santa Maria alle Scotte, Viale Bracci, 14, 53100 Siena, Italy; ^4^Department of Veterinary Medicine, University of Perugia, Via San Costanzo, 06123 Perugia, Italy; ^5^Department of Veterinary Medicine, University of Milan, Via dell'Università 6, 26900 Lodi, Italy

## Abstract

The aim of this research was to evaluate the inflammatory and/or oxidative damage related to silver nanoparticles (AgNPs), which are responsible for negative effects on sperm physiology and metabolism. Thirty New Zealand White rabbit bucks were divided into 5 experimental groups (6 animals/group): *Control*, treated with 0.9% NaCl; *AgNP*, treated with a 5 mM AgNP solution; *LPS*, treated with 50 g/kg b.w. *E. coli* LPS; *AgNPs + NSAID*, treated with an anti-inflammatory drug at 0.2 mg/kg b.w. and 5 mM AgNPs; and *AgNPs + Vit E*, treated with 0.18 mg/kg b.w. vitamin E and 5 mM AgNPs. Sperm quality and oxidative and inflammatory status were assessed at different times (0-60 days). Two statistical models were built: the first evaluated the effects of AgNPs and LPS (vs. Control), whereas the second evaluated the protective effect of an NSAID and vitamin E against AgNP-induced damage. Three principal component analyses were performed: *sperm traits* (motility, volume), *oxidative status* (antioxidants, oxidative metabolites, and redox reactions), and *cytokines* (TNF-*α*, IL-8, and IL-6). A negative effect on reproductive traits resulted after NP administration. In particular, an inflammatory/oxidative response took place in the reproductive tract during the first 2-3 wks of AgNP administration (*cytokine* and o*xidative metabolite* generation); the inflammatory/oxidative thrust impaired the status of rabbit tissues (seminal plasma, sperm, and blood), inducing a response (increased *antioxidant enzymes* and *redox reactions*) at 4-7 wks; oxidative stress, if not totally counteracted, likely induced toxicity in the late phases of AgNP administration (8-9 wks). In conclusion, exposure to silver nanoparticles produced a similar but more persistent effect than that of LPS on rabbit reproductive tissues: AgNP administration triggered a proinflammatory response linked to oxidative thrust, worsening many sperm parameters. However, only anti-inflammatory treatment counteracted the negative effects of AgNPs, whereas vitamin E seemed to act as an adjuvant, attenuating the oxidative cascade.

## 1. Introduction

Silver nanoparticles (AgNPs), one of the most popular nanomaterials, are commonly used in consumer products and biomedical devices despite their potential toxicity. The increasing use of AgNPs has raised concerns about their potential risks to human health. The majority of reproductive and developmental toxicity studies of AgNPs have been performed via oral exposure, while others used intravenous, intraperitoneal, and subcutaneous injections and intratracheal instillation [1]. Dose levels and pharmacological form are the most critical factors affecting AgNP toxicity; moreover, the toxicity of AgNPs may vary with the rate and degree of the dissolution of AgNPs, which depend on particle size, surface functionalization, crystallinity, concentration, and temperature [2].

A developmental neurotoxic effect of AgNPs was reported in mice and rats [3]. In laboratory animals, AgNP exposure was associated with testicular/sperm toxicity in males and ovarian and embryonic toxicity in females [1]. However, there is still limited knowledge on the effects of AgNPs on spermatogenesis. In rodents, testicular histology and sperm parameters are linked; thus, alterations in testicular structure are usually accompanied by alterations in testicular function. *In vitro* and *in vivo* studies stated that NPs are able to cross the blood-testis barrier (BTB). The BTB is a testis-specific ultrastructure between adjacent Sertoli cells that controls the movements of germ cells across the seminiferous epithelium [4]. The transport of AgNPs through the BTB was reported in male mice [5, 6], rats [5], and rabbits [7]. In our previous study [8], using rabbits as an animal model [9], we intravenously administered (0.6 mg/kg b.w.) AgNPs (average size: 45 nm) to evaluate reproductive activity and sperm quality. These results showed that AgNPs reach the testes, compromising sperm motility, sperm speed, and acrosomal and mitochondrial shape and function. Transmission electron microscopy (TEM) analysis did not show any evident morphological damage in the testes; however, AgNPs were visible in spermatids and ejaculated sperm.

Reactive oxygen species (ROS), which cause oxidative stress and inflammation, are hypothesized to be major causative factors in the toxicity of nanomaterials [10]. Oxidative stress occurs when the ROS levels exceed the body's antioxidant defense systems; thus, antioxidants play a major role in the prevention of oxidative stress. It may be limited by chain-breaking antioxidants like vitamins E and C that can be taken as drugs [11]. Actually, ROS are free radicals that extraordinary importance in many stages required for normal fertilization, such as sperm capacitation, hyperactivation, and spermatocyte fusion [12]. However, excessive production of ROS causes sperm dysfunction through lipid peroxidation, motility loss, and DNA damage. Vitamin E is also known to be scavenger antioxidants, it is thought that it may suppress the production of ROS and reduce the production of nitric oxide (NOS) [13].

Bacterial lipopolysaccharide (LPS), an active component of the Gram-negative bacterial cell wall, has been reported to be a useful model for studying the effect of inflammation on reproductive functions [14, 15] and, in particular, spermatogenesis [16]. The application of LPS could clarify the molecular mechanisms involved in the inflammatory process in the testis and explain the effects of some therapeutic options.

Nonsteroidal anti-inflammatory drugs (NSAIDs) are among the most consumed drugs in the world due to their role as analgesic, anti-inflammatory, and antipyretic agents [17].

In this context, the aim of the study was to explain the role of inflammatory and oxidative damage related to AgNPs, which are responsible for negative effects on sperm physiology and metabolism because they alter the BTB. Rabbit bucks were alternatively treated with LPS and AgNPs to induce a response and with NSAIDs and vitamin E to counteract this response; sperm quality and oxidative and inflammatory status were assessed at different times after AgNP administration.

## 2. Material and Methods

### 2.1. AgNP Characterization

AgNPs were primarily characterized by UV-visible spectroscopy. Ultraviolet-visible (UV-vis) spectra were obtained using WPA (Mechasys, South Korea). Particle size distribution analysis was carried out using DLS by a Zetasizer Nano ZS90 instrument (Malvern Instruments, Ltd., UK). XRD patterns were obtained using an X-ray diffractometer (Bruker D8 DISCOVER, Bruker AXS GmBH, Karlsruhe, Germany). A transmission electron microscope (JEM-1200EX) was used to determine the size and shape of the AgNPs. The NPs were characterized according to a previously described method [7].

### 2.2. Chemicals and Preparation of AgNPs and LPS

Every reagent, unless otherwise specified, was provided by Merck KGA (Darmstadt, Germany).

Stock solutions and samples were diluted with deionized water. AgNPs were kindly provided by Cericol Colorobbia Research Center, Sovigliana Vinci, Firenze (Italy). The NP suspension was prepared by polyol-mediated synthesis using a bottom-up approach and an organometallic silver precursor. By employing this procedure, metal NPs could be dispersed in water by means of metallic salt nucleation. The process involved an initial phase in which reagents were added in a high-temperature environment to a reaction mixture containing a solvent, such as glycolic solvent medium, with a high capacity for complexing. The next step was the crystal growth phase, which was performed by mixing the reagents, including a surfactant such as polyvinylpyrrolidone (PVP), at room temperature. The obtained NPs were stable for long periods of time without showing aggregation or changes in chemical characteristics. The concentration of the stock solutions of NPs was 1% in distilled H_2_O with PVP (<1%) as a stabilizing agent.

The LPS solution was prepared from an *Escherichia coli* LPS (*E. coli*; 0127:B8, Sigma–Aldrich) analytical reagent at a concentration of 50 *μ*g/kg body weight (b.w.) and dissolved in 2 ml of a saline solution following the suitable LPS dose for the induction of a reversible inflammatory state that was previously tested in rabbit bucks [16].

### 2.3. Animals, Housing Conditions, and Approval of the Experimental Procedures

New Zealand White male rabbits selected for their high semen quality and high repeatability were enrolled in this study. Rabbits were housed in the experimental facility of the University of Siena. Semen was collected with an artificial vagina filled with warm water at approximately 38°C. Animals were housed in individual cages with a photoperiod of 16 h light/day, at an intensity of 40 lux, and at temperatures ranging from 16 to 25°C. Fresh water was always available. The rabbits were fed *ad libitum* with a standard diet composed of dehydrated alfalfa meal (40%), soybean meal (18%), barley (30%), wheat bran (10%), and minerals and vitamins (2%). The feed, purchased from Mignini s.p.a. (Petrignano, Italy) as pellet chow, had the following chemical composition: 17.5% crude protein, 15.5% crude fiber, 2.5% fat, and 6.2% ash.

At 140 days of age, rabbits were trained for semen collection for approximately 2 wks. During the training period, libido (defined as the time between the introduction of a female rabbit and ejaculation) and several sperm traits of each rabbit buck were analyzed.

This study was conducted in accordance with the Guiding Principles in the Use of Animals in Toxicology and was approved by the Animal Ethics Monitoring Committee of the University of Siena (CEL AOUS 21.10.09).

### 2.4. Study Design

To investigate the effect of AgNPs on the reproductive traits and inflammatory state of rabbits, 30 New Zealand White rabbit bucks of the same age (8 months) and weight (approximately 4.3 ± 0.8 kg) were divided into 5 experimental groups (6 animals/treatment):

- C*ontrol*, treated with 2 ml of a saline solution (0.9% *w*/*v* NaCl)
- *AgNPs*, treated with a 5 mM AgNP solution (0.6 mg/kg b.w.)
- *LPS*, inoculated with 2 ml of 50 g/kg b.w. *E. coli* LPS (0127:B8, Sigma–Aldrich, Munich, Germany)
- *AgNPs + NSAID*, treated with an anti-inflammatory agent (METACAM 2 mg/ml, active principle: meloxicam; injectable solution; Boehringer Ingelheim, Milano, Italy) at 0.2 mg/kg b.w. for 3 consecutive days and a 5 mM AgNP solution (0.6 mg/kg b.w.)
- *AgNPs + vitamin E* (*AgNPs + vit E*), treated with an antioxidant (VITALENE E: 50 mg/ml D-alfa-tocopheryl acetate, i.m. injectable solution, Fatro Industria Farmaceutica Veterinaria s.p.a., Ozzano dell'Emilia, Italy) at a dosage of 0.18 mg/kg b.w. daily for 3 consecutive days.

The AgNP dose was chosen based on a previous dose-response study [7]. The 0.6 mg/kg b.w. dose has been considered the minimum quantity, which determines the NPs detection in the rabbit semen and effective reproductive outcomes.

For the saline solution, LPS and AgNP treatments, a volume of 2.0 ml was injected intravenously into the marginal vein of the right ear of rabbits. *NSAID* and vitamin E administration was provided with an intramuscular injection on the right side of rabbits. When concomitant intravenous administration was performed, rabbits were administered a single injection into the same marginal ear vein.

During this trial, the health status of rabbits was assured daily by monitoring feeding and drinking behavior, live weight, and body temperature.

### 2.5. Semen, Blood, and Organ Sampling

**Figure 1:**
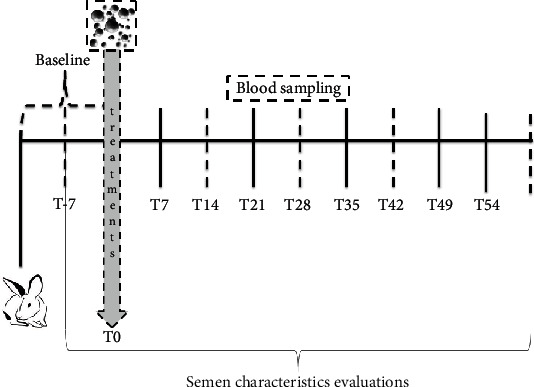
Experimental design. The training of animals for semen collection lasted two weeks. Baseline values of semen were established from ejaculated samples collected one week before the start of treatment. Thereafter, seminal traits were analyzed weekly.

Semen samples were collected weekly from each rabbit buck (Figure 1) starting one week before the start of the trial and during the experimental period of 60 days, for a total of 11 samplings. Semen samples were collected by means of an artificial vagina heated to 38°C with water, and samples were immediately transferred to the laboratory [18]. The evaluations of sperm quality were immediately performed on raw samples as described in “Sperm Quality Assessment.” The samples resulting from the quality evaluation were divided into two aliquots of 10^8^/ml: one was centrifuged (at 2500 rpm for 20 min) to obtain seminal plasma and washed spermatozoa, and the other was stored at -80°C for the analysis of oxidative status (see “Oxidative Status of Sperm, Seminal Plasma, and Blood” and “ Cytokine Assessment in Seminal Plasma”).

Every week, blood samples (2 ml) were taken from the auricular marginal vein after the local application of an anesthetic cream (EMLA®, Codifa, Milan, Italy) using a 2.5 ml syringe fitted with a butterfly needle (G23). Plasma was obtained from blood samples collected in tubes containing Na_2_-EDTA and immediately centrifuged at 2500 × *g* for 15 min at 4°C.

### 2.6. Sperm Analytical Determinations

#### 2.6.1. Sperm Quality Assessment

After collection, the semen was immediately subjected to the following analyses:

1. *Volume* (ml), which was determined by graduated tubes
2. *Sperm concentration* (number of sperm × 106/ml), which was measured by means of a Thoma–Zeiss cell counting chamber with a ×40 objective lens
3. *Live spermatozoa* (%), which were measured using the eosin/nigrosine exclusion test
4. *Kinetic characteristics* were analyzed by a computer-assisted semen analyzer (model ISAS®4.0, Valencia, Spain) after appropriate dilution (1/20) with a Tyrode's albumin lactate pyruvate buffer at pH 7.4 and 296 mOsm/kg. This system consisted of a negative phase-contrast optic system (Olympus CH-2) equipped with a Sony CCD camera. The setup parameters were established previously, and the acquisition rate was set at 100 Hz [19]. For each sample, two drops and six microscopic fields were analyzed for a total of 300 spermatozoa. The recorded sperm motion parameters were motility rate (percentage of motile sperm/total sperm) and curvilinear velocity (VCL, *μ*m/s: the sum of the incremental distances moved by the sperm in each frame along the sampled path divided by time).

#### 2.6.2. Oxidative Status of Sperm, Seminal Plasma, and Blood

The extent of lipid peroxidation induced in the sperm membrane, SP, and blood plasma was assessed by measuring the malondialdehyde (MDA) content. The nitric oxide (NO) concentration and catalase (CAT) and glutathione peroxidase (GPX) enzyme activities were also evaluated in both sperm and blood samples.

Sperm cells were suspended in 1 ml of phosphate-buffered saline (PBS). The sperm specimens were lysed through rapid freeze-thawing at -80°C and +35°C three times. Finally, the samples were centrifuged at 2500 × *g* for 5 min and divided into aliquots for MDA, NO, CAT, and GPX determinations. The aliquots were stored at -80°C until analysis.

The lipid peroxidation in lysed sperm cells and in the blood plasma of rabbits was assessed by determining MDA levels. A total of 0.4 ml of 0.04 M Tris-HCl and acetonitrile containing 0.1% butylated hydroxytoluene (BHT) was added to 0.4 ml of lysed sperm cells or blood plasma. After derivatization with 2.4 ml of dinitrophenylhydrazine according the method of Shara et al. [20] with minor modifications, the samples were immediately stirred, extracted with 5 ml of pentane, and dried using nitrogen. MDA hydrazone was quantified by isocratic high-performance liquid chromatography using a Waters 600 E system controller HPLC instrument (Milford, MA, USA) equipped with a Waters Dual *λ* 2487 UV detector (Milford, MA, USA) set at 307 nm. A 5 *μ*m ultrasphere ODS C18 column (Beckman, San Ramon, CA, USA) was used with a mobile phase composed of acetonitrile (45%) and HCl 0.01 N (55%) at a flow rate of 0.8 ml/min. A calibration curve with concentrations of MDA ranging from 0.2 to 10 nmol/ml was used for MDA quantification. The MDA concentration was calculated by peak areas using an Agilent 3395 integrator (Agilent Technologies, Santa Clara, CA, USA). The results were expressed as nmol/10^6^ sperm cells or nmol/ml blood plasma.

The MDA content was also assessed in the seminal plasma of samples following the method reported by Mourvaki et al. [21]. The molar extinction coefficient of MDA was 1.56 × 10^5^ 1/Mcm. The results are presented as nmol MDA/ml.

The NO concentration was measured as total nitrates and nitrites (NO_2_ + NO_3_) by the Griess reaction method [22]. A colorimetric assay kit (Cayman Chemical Company, Ann Arbor, MI, USA) was used to measure the total nitrate/nitrite concentration in a two-step process. The first step was the conversion of nitrate to nitrite utilizing nitrate reductase. The second step was the addition of Griess reagents, which converted nitrite into a deep purple azo compound. Briefly, 80 *μ*l of lysed sperm cells or 40 *μ*l of blood plasma was mixed in triplicate with 20 *μ*l of nitrate reductase and incubated for one hour. After the incubation time, 100 *μ*l of the Griess reagent was added. The absorbance of the purple chromophore was measured at 540 nm using a microplate reader from Bio-Rad Laboratories Inc. (Hercules, CA, USA). Nitrite concentrations were calculated from a standard curve constructed using a sodium nitrite stock solution provided by the kit. The results were expressed as nmol/10^6^ cells (spermatozoa) or as *μ*M (blood plasma).

CAT activity was measured with a microassay procedure described by Johansson and Borg [23]. Briefly, 100 *μ*l of spermatozoa lysate or blood plasma was added to an equal volume of ice-cold phosphate buffer (0.125 M, pH 7.4) containing 1 mM EDTA and then centrifuged at 4000 × *g* for 15 minutes at 4°C. The analyses were performed in triplicate, and one unit of catalase activity was defined as the amount of enzyme that would cause the formation of 1 nmol of formaldehyde per minute at 25°C. The results were expressed as U/10^6^ cells or U/ml.

GPX activity was evaluated as described by Flohé et al. [24]. Briefly, 100 *μ*l of lysed sperm cells or blood plasma was diluted (1 : 1) in cold 0.25 M sucrose in 0.1 M phosphate buffer (pH 7.4) and immediately centrifuged at 40000 × *g* for 20 min at 4°C. The enzymatic activity was evaluated spectrophotometrically (Perkin Elmer Lamda35) by measuring the change in absorbance at 340 nm produced by oxidation of NADPH. One unit of GPX activity was defined as the amount of enzyme that oxidized 1 *μ*mol of NADPH at 37°C per min. The analyses were performed in triplicate, and the enzymatic activity was expressed as U/10^6^ cells or U/ml.

#### 2.6.3. Cytokine Assessment in Seminal Plasma

A panel of cytokines (IL-6, IL-8, IL-1b, and TNF) was detected and quantified in seminal plasma using the Bio-Plex Cytokine assay (Bio-Rad Laboratories S.r.l., Segrate, Milano, Italy) following the manufacturer's protocols and the method reported by Han et al. [25]. Briefly, 96-well plates were prewetted with 200 ml of assay buffer (provided by the manufacturer) for 10 minutes and then aspirated using a vacuum manifold. Standards and seminal plasma (25 ml) were added to appropriate wells, followed by the addition of assay beads. The plates were incubated at RT for 30 minutes with mild agitation; the fluid was then removed by vacuum, and the wells were washed twice with wash buffer. Detection antibodies were added to each well and incubated for 1 hour at RT. The fluorescent conjugate streptavidin–phycoerythrin was added to each well, and plates were incubated for 30 minutes at RT. The fluid was then removed by vacuum, and the wells were washed twice. Analysis of each sample was performed in duplicate. The limit of sensitivity was 1.95 pg/ml, and the linear range of detection was 1.95 to 32.000 pg/ml for all the cytokines analyzed in this study. Potential interference of seminal plasma was tested by running parallel standard curves without seminal plasma. Data were collected and analyzed using a Bio-Plex 200 instrument equipped with BioManager analysis software (Bio-Rad).

### 2.7. Statistical Analysis

The data were analyzed using linear mixed models (LMMs) in which animals and days were included as subjects and repeated factors, respectively, with a first-order autoregressive covariance structure. Two models were built, and both evaluated the effect of group (3 levels), time (10 levels), and their interaction.

The first model evaluated the effects of AgNPs and LPS (against the control).

The second model evaluated the possible protective effect of NSAIDs and vitamin E (the AgNPs + NSAID and AgNPs + vit E groups, respectively) on AgNP-induced damage (AgNP group). Sidak adjustment was used for carrying out multiple comparisons. Diagnostic graphics were used for testing assumptions and outliers. Logarithmic transformation was used for TNF-*α* determination.

Then, we performed principal component analysis (PCA) to reduce traits according to *sperm traits* (PCA1), *oxidative status* (PCA2), and *cytokines* (PCA3).

Variables were included in PCA after inspection of the correlation matrix to identify very high correlations [26, 27]. Moreover, the Kaiser-Mayer-Olkin (KMO) measure of sample adequacy and the Bartlett test of sphericity were conducted. Static sperm and sperm catalase were not included as highly correlated with motility rate and seminal plasma catalase, respectively.

Principal components (PC) showing eigenvalues > 1 were retained and rotated using the varimax method. The PC label was chosen based on items that had structure coefficients higher than |0.5| [28, 29]. The scores were calculated for each PC using the regression method; then, they were normalized by scaling between 0 and 1. Finally, these PCs were evaluated by the two LMMs. Statistical analyses were performed with SPSS Statistics version 25 (IBM, SPSS Inc., Chicago, IL, USA). Statistical significance was set at *P* ≤ 0.05.

## 3. Results

### 3.1. LMM Analysis

First, the effects of AgNPs, LPS, the NSAID, and vitamin E on individual variables were assessed (Tables S1 and S2). Almost all changes in analyzed variables were statistically significant in the experimental groups (except the MDA content in sperm and IL-8 content in the SP of the AgNPs vs. AgNPs + NSAID vs. AgNPs + Vit E treatment groups) for both time and treatment. Consequently, multivariate analysis was performed to evaluate the results.

### 3.2. Principal Component Analysis

**Figure 2:**
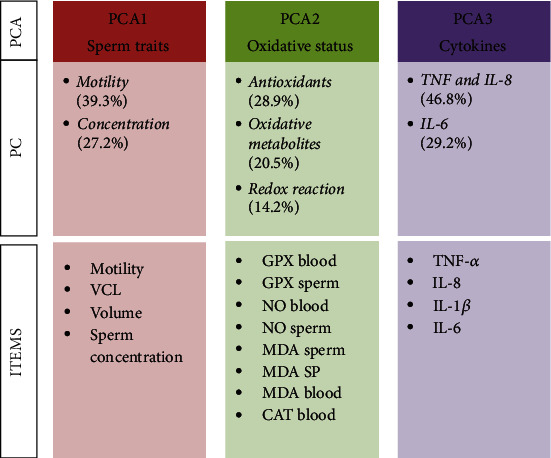
Scheme of principal component analysis (% of variance explained by the resulting PC). From the 16 initial variables (ITEMS), 7 variables were selected (PC: motility, volume, antioxidants, oxidative metabolites, redox reactions, TNF IL-8, and IL-6) in the three PCAs (sperm traits, oxidative status, and cytokines). PCA: principal component analysis; PC: principal component; VCL: curvilinear velocity; GPX: glutathione peroxidase; NO: nitric oxide; MDA: malondialdehyde; CAT: catalase; SP: seminal plasma; TNF-*α*: tumor necrosis factor alpha; IL: interleukin.

Three PCAs were selected in accordance with the biological meaning of these variables (Figure 2): PCA1 described changes in *sperm traits* (motility, volume), PCA2 described changes in *oxidative status* (antioxidants, oxidative metabolites, and redox reactions), and PCA3 described changes in *cytokines* (TNF-*α*, IL-8, and IL-6).

In this way, we reduced the number of variables while retaining the ability to describe different phenomena in detail. From the 18 initial variables (Tables S1 and S2), 16 variables were subjected to PCA after inspection of the correlation matrix (Table 1, Figure 2).

The total variances explained by the analyses ranged from 63.3% for PCA2 to 76.0% for PCA3 (Figure 2). The KMO and Bartlett tests confirmed the sample adequacy and absence of identity matrices (*P* < 0.001).

The first PCA yielded two PCs, of which the first had high loadings for motility, while the second was positively correlated with semen volume and negatively correlated with sperm concentration.

The second PCA yielded three PCs: on PC called “*Antioxidants*” included the GPX level in both the spermatozoa and blood plasma, the PC “*Oxidative metabolites*” had high loadings for blood plasma NO and MDA contents (in both the spermatozoa and seminal plasma), and the PC “*Redox reaction*” was negatively correlated with blood plasma catalase activity and positively correlated with sperm NO content.

Finally, the cytokines were separated into two PCs: the first had the highest loadings for TNF-*α* and IL-8 (and low for the IL-1*β*), and the second only had the highest loadings for IL-6 (PCA3).

### 3.3. Effects of AgNP and LPS Treatments: Sperm Traits, Oxidative Status, and Cytokines

After normalization of the scores, the seven PCs were analyzed by LMMs. The first LMM evaluated the effects of AgNPs by comparing them with an LPS-induced inflammation model. The AgNP and LPS treatments affected all parameters except volume (Table 2).

Both the AgNP and LPS treatment groups had lower marginal mean scores for the *Antioxidant* PC than that of the control and higher scores for *Redox reaction*, *TNF-α and IL-8*, and *IL-6* PCs (*P* < 0.001). The differences in marginal means between the two treated groups (AgNPs vs. LPS) were not significant for these PCs. Conversely, the AgNP group had the lowest marginal mean score for *Motility* PC (*P* < 0.01) and the highest for the *Oxidative metabolite* PC (*P* < 0.01).

**Figure 3:**
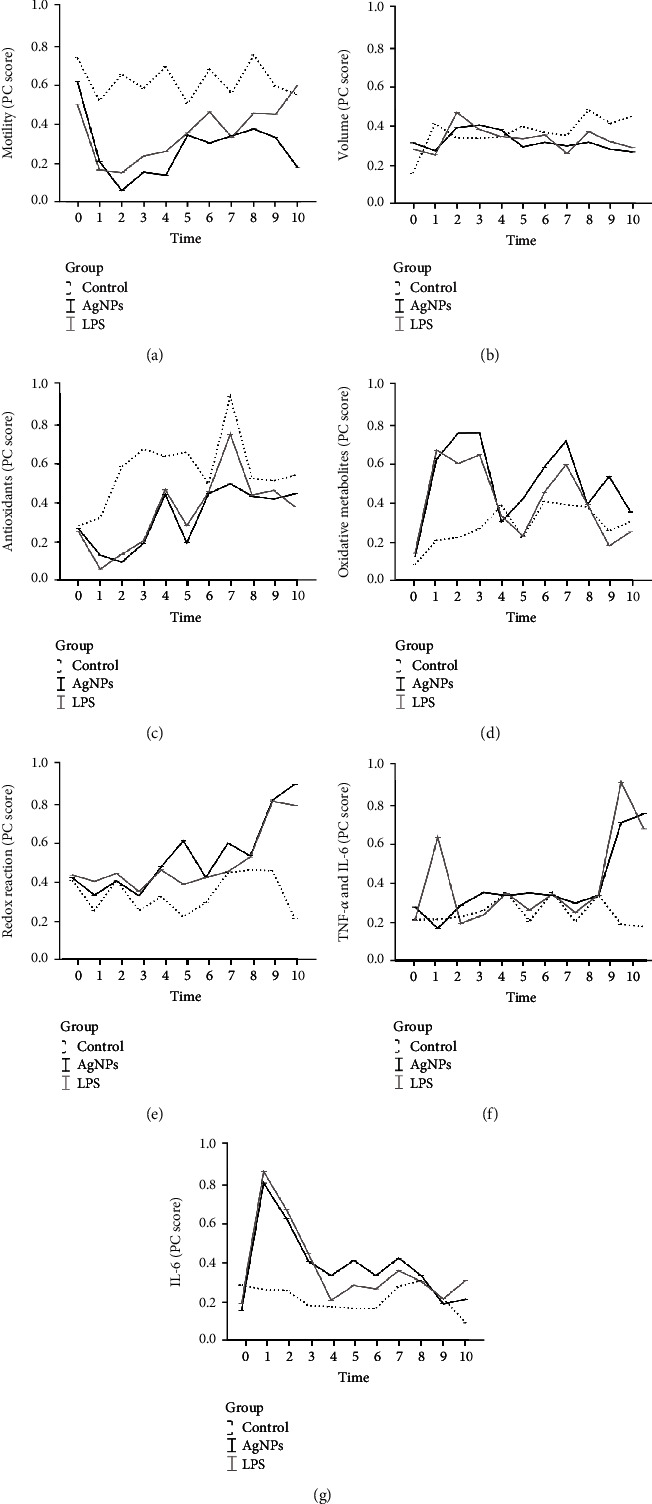
Effects of LPS and AgNPs on the PC scores indicating sperm motility (a), volume (b), oxidative status (c–e), and cytokines (f, g). Predicted values and standard error of PC scores by models evaluating the effects of groups (Control, AgNPs, and LPS), time (10 levels), and their interaction. Control group: treatment with 2 ml of a 0.9% saline solution; AgNP group: treatment with a 5 mM AgNP solution; LPS group: inoculated with 50 g/kg b.w. *E. coli* LPS.

*Motility* scores decreased in the AgNP group after 3 days (*P* < 0.001) and, unlike the LPS group, remained lower than those of the control group during the entire period (*P* < 0.05; Figure 3(a)).

After 3 days, the AgNP and LPS groups showed lower *Antioxidant* scores than that of the control group (*P* < 0.01) and higher *Oxidative metabolites* scores (*P* < 0.001) (Figures 3(c) and 3(d)). These trends reversed in these groups after 21 d (T4; *P* < 0.001). Afterwards, the scores of the PCs *Antioxidants* and *Oxidative metabolites* followed a fluctuating and specular trend. However, the AgNP group showed higher values of *Oxidative metabolites* than those of the control at almost all time points (except at T4, T8, and T10; *P* < 0.001), while the LPS group did not differ from the control from T4 until the end of the period.

Compared to those of the control, *Redox reaction* scores began to increase in the two treated groups after 21 days (T3; *P* < 0.01). Finally, the AgNP group had the highest values (*P* < 0.05; Figure 3(e)).

The LPS group showed peculiar changes in the *TNF-α and IL-8* scores (Figure 3(f)), which increased after 3 days (*P* < 0.001) but remained constant in the AgNP group for 49 days. However, in the last two weeks of observation, both the AgNP and LPS groups had higher *TNF-α and IL-8* scores than those of the control group (*P* < 0.001).

The pattern of *IL-6* cytokine was similar in the two treated groups (Figure 3(g)): in both groups, a peak after 3 days (*P* < 0.001) and a subsequent decline were observed (*P* < 0.01). Then, *IL-6* scores were higher in both the AgNP and LPS groups than in the control at the last time point (*P* < 0.05).

### 3.4. Effects of an NSAID and Vitamin E on AgNP-Induced Inflammation: Sperm Traits, Oxidative Status, and Cytokines

**Figure 4:**
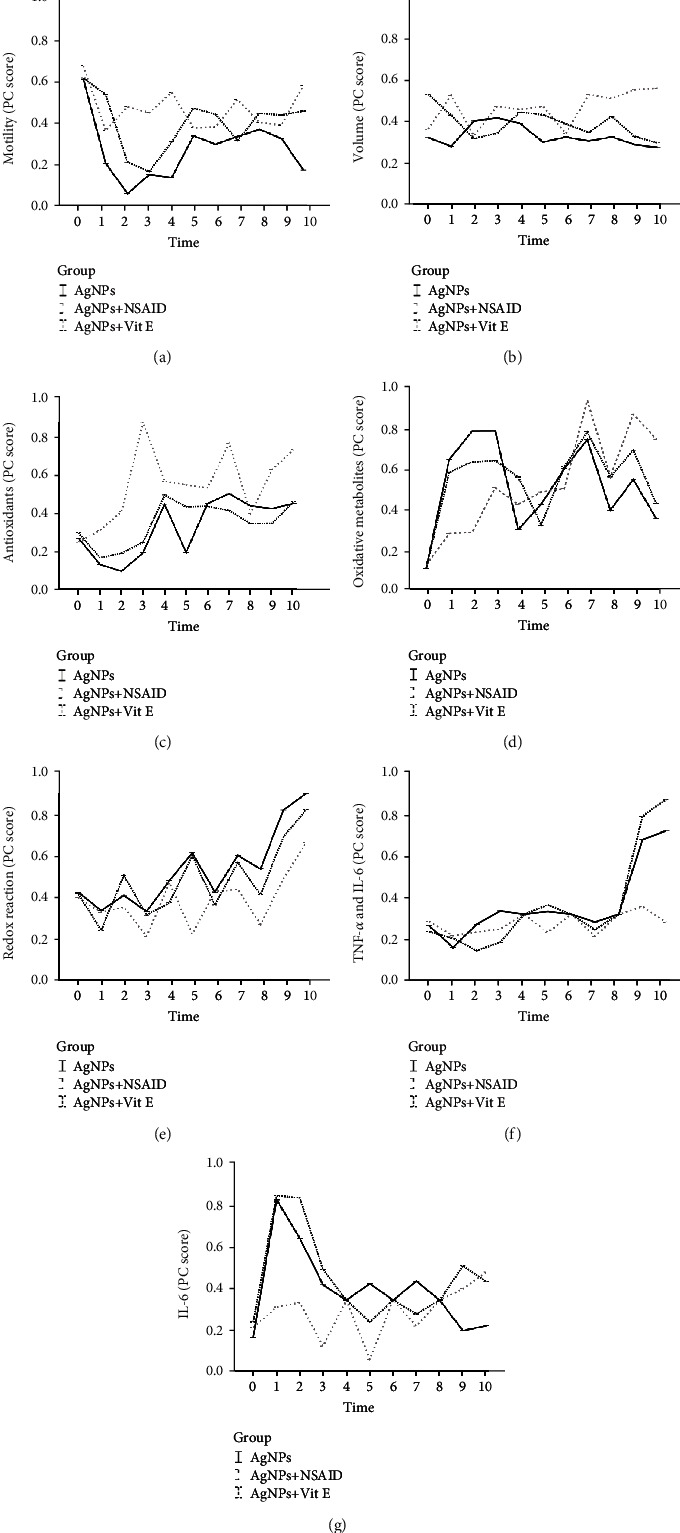
Effects of an NSAID and Vit E on AgNP-induced inflammation and scores indicating sperm motility (a), volume (b), oxidative status (c–e), and cytokines (f, g). Predicted values and standard error of PC scores by models evaluating the effects of groups (AgNPs, AgNPs + NSAID, and AgNPs + Vit E), time (10 levels), and their interaction. AgNP group: treatment with a 5 mM AgNP solution; AgNPs + NSAID group: treatment with 0.2 mg/kg b.w. anti-inflammatory agent and a 5 mM AgNP solution; AgNPs + Vit E group: treatment with 0.18 mg/kg b.w. *α*-tocopheryl acetate and a 5 mM AgNP solution.

The second LMM (Table 3 and Figure 4) evaluated the effects of the NSAID and vitamin E on the inflammation induced by nanoparticles (AgNP group). The addition of the NSAID increased the marginal means of *Motility* (*P* < 0.05), *Volume* (*P* < 0.05), and *Antioxidants* (*P* < 0.001) while reducing *Redox reaction* (*P* < 0.001) and cytokine (*P* < 0.001) scores compared to those of both the AgNP and AgNPs + Vit E groups.

The AgNPs + Vit E group showed intermediate values for *Motility* and *Redox reaction* (*P* < 0.05) scores, but its *Volume* and cytokine PC scores did not improve compared to those of the AgNP group. No difference between groups was found between the marginal *Oxidative metabolite means* (Table 3).

The administration of the NSAID increased after 1 wk, and they remained higher than those of the AgNP group at most time points (T2-T4, T7, and T10; P <0.05; Figure 4(a)). The AgNPs + Vit E group had intermediate *Motility* scores: an initial drop, similar to the AgNP group, was observed (*P* < 0.001), but during the last few weeks, the scores were the same as those of the AgNPs + NSAID group (Figure 4(a)). Differences in *Volume* between groups were only observed during the last 3 weeks, when the administration of the NSAID improved the *Volume* scores (*P* < 0.05; Figure 4(b)).

The AgNPs + NSAID group had higher *Antioxidants* scores than those of the control at all time points (*P* < 0.05) except at T8. Conversely, higher *Antioxidants* scores in the AgNPs + Vit E group than in the control group were only observed at day 35 (T5; *P* < 0.001; Figure 4(c)). NSAID administration delayed the increase in the *Oxidative metabolite* score, maintaining lower scores than those of the other two groups for the first 3 weeks (*P* < 0.05; Figure 4(d)). Indeed, the *Oxidative metabolites* trend of the AgNPs + NSAID group was similar to that of the group without induced inflammation (control group, Figure 3(d)). Vit E administration mitigated this initial increase in *Oxidative metabolites* compared to the control group (*P* < 0.05), although the scores were higher than those of the AgNPs + NSAID group (*P* < 0.05; Figure 4(d)). *Redox* reaction scores fluctuated even if the AgNPs + NSAID group showed the lowest values at different time points (T3, T5, and T7; *P* < 0.05). In the last 3 weeks, the score increased in all groups in the following order: AgNPs > AgNPs + Vit E > AgNPs + NSAID (Figure 4(e)).

NSAID administration increased cytokine scores (Figures 4(f) and 4(g)), so this group showed almost the same trends as those of the control group (Figure 3(g)). The trends of the group receiving vitamin E were similar to those of the AgNP group.

## 4. Discussion

Silver nanoparticles (AgNPs) are emerging as one of the most commonly used nanomaterials [30]: they are widely used as antibacterial agents in biomedical and consumer goods [31]. For these reasons, they could end up in the environment or accumulate in the organs of animals and humans [32]. Our results demonstrated that AgNPs negatively interfere with the reproductive tissues of male rabbits through an inflammatory/oxidative mechanism similar but more persistent than that of LPS. This hypothesis was confirmed by the positive effect of vitamin E administration and more so by administration of an anti-inflammatory drug (NSAID) on semen traits.

It is widely known [16, 33] that LPS negatively affects semen parameters; however, the present results showed that the recovery time of LPS-exposed rabbits was shorter than that of AgNP-exposed rabbits because NP administration reduced the speed and the number of motile cells for more than two spermatogenic cycles [7, 8]. Similarly, all parameters except sperm concentration were negatively and more affected by AgNPs than by LPS administration.

It was likely that the persistence of the AgNP effect was related to testicular damage (structural alteration of germinal cells and disruption of the gap junction) more severe to that found in rabbits after LPS administration [34]. Indeed, normal spermatogenesis was restored only approximately 50 days after AgNP administration, as shown by sperm motility, which returns to standard conditions after 7 weeks. Whether AgNPs accumulated in the sperm during transit in the epididymis and/or via accessory glands remains to be determined.

Body of literature reported that AgNP administration is conveyed in the liver, spleen, and kidneys of male mice, but elevated concentrations of AgNPs were also detected in the testes (1/3 of the level administrated) 4 months after the last injection of AgNPs [6]. Indeed, we found higher concentration of AgNPs in semen for a long time (0.43 and 0.29 ng/ml, respectively, after 15 and 60 days from administration).

In mice [6], rats [35], and rabbits [7], it was reported that AgNPs cross the BTB and destroy gap junction. The gap junction is an important mechanism for maintaining and regulating cell differentiation, tissue physiology, and the normal function of reproductive organs [36]. This tendency was likely due to the persistence of AgNPs in the testes for a long time.

This evidence suggested that a cooperative effect due to both inflammatory and oxidative thrust may trigger damage during spermatogenesis because the AgNPs crossed the BTB.

Although our data do not reveal in detail how AgNPs affected sperm traits, we could hypothesize different mechanisms of action based on the occurrence of effects. Three temporary phases may have occurred:

1. An inflammatory/oxidative response (see *cytokine* and o*xidative metabolite* generation; Figures 3(f) and 3(g) and Figure 3(d), respectively) took place during the first 2-3 weeks after AgNP administration (from T0-1 to T2-3)
2. The inflammatory/oxidative thrust impaired the health status of reproductive tissues (seminal plasma, sperm, and blood), inducing a subsequent antioxidant response (increased *antioxidant enzyme* and *redox reaction* levels; Figures 3(c) and 3(e), respectively) between 4 and 7 weeks, where the reproductive tissues (seminiferous tubules, Sertoli cells, and epididymis) partially improved the environmental conditions and stabilized them
3. Oxidative stress, when not totally counteracted, could induce reduction of sperm traits (see *Motility*; Figure 3(a)) and cytotoxicity through DNA damage in the late phases (T8-10).

Under normal circumstances, the body has immune and antioxidant systems to counteract oxidative stress. However, under extreme oxidative challenge, such as those detected upon nanoparticle exposure [37], the antioxidant mechanisms can become overwhelmed, and only the administration of antioxidants (e.g., vitamin E administration) or an anti-inflammatory drug (NSAID) could positively reduce oxidative damage.

The proinflammatory/oxidative effect induced by AgNPs on sperm traits was also confirmed by the trend of proinflammatory cytokine and oxidative metabolite (NO and MDA). Cytokine levels in the seminal plasma increased similarly in the AgNP and LPS groups, with a reduction due to NSAID administration. In particular, the IL-6 levels showed a peak a few days after AgNP injection as a result of the acute inflammatory response [38], whereas the IL-8 level increased in the last 20 days of the experiment, suggesting chronic inflammation in rabbit reproductive tissues. Eggert-Kruse et al. [39] found higher IL-8 concentrations in seminal plasma of hypofertile men than in control ones, suggesting that the IL-8 level is inversely correlated with sperm kinetic parameters. In addition, literature studies reported that testicular inflammation upregulates IL-1*β*, IL-1*α*, IL-6, and TNF-*α*, which induce adverse effects on germ cells [40].

Pérez et al. [41] described a positive correlation between IL-6 levels in rats and tight junction impairment. The increased generation of IL-6 also accompanied increased ROS production during endotoxic shock.

Based on these findings, it may be hypothesized that the increased oxidative stress induced by LPS and AgNPs amplified the production of certain cytokines, specifically, IL-8 and IL-1*β*, which in turn resulted in the recruitment of additional neutrophils and increased the generation of ROS and NOS. Because of the low scavenging system of sperm, the generated oxygen radicals augmented the injury further.

The *Oxidative metabolite* (MDA and NO) scores were higher in the LPS and AgNP groups than in the control group. In both treated groups, it was possible to see maximum oxidation at 3 and 42 days (T0-1 and T7) and a subsequent reduction. This trend was related to the high oxidative thrust induced by reactive radical (ROS, NOS) production from reproductive system.

In agreement, in our previous study [42], we reported higher values of reactive oxygen metabolites (ROMs) in semen from LPS-treated rabbits than in control rabbits, particularly a few days after administration. Similarly, Rezazadeh-Reyhani et al. [43] found a dose-dependent increase in mouse testicular and sperm NOS contents with AgNP administration (5 mg/kg) after 35 days.

The present oxidative trend confirmed the correlation among excessive radical generation, lipid peroxidation, and damage to the sperm membrane. Mammalian spermatozoa are extremely vulnerable to free radical attack and the induction of a lipid peroxidation process that disrupts the integrity of the plasma membrane and impairs sperm motility [44, 45]. This susceptibility resides in the presence of targets for free radical attack in sperm cells, as the abundance of polyunsaturated fatty acids (almost 50% are long-chain polyunsaturated fatty acids [21, 46]) in the plasma membrane, which is necessary for sperm movement and for the membrane fusion events associated with fertilization. Unfortunately, such high fatty acid unsaturation renders sperm cells particularly prone to oxidative attack because the conjugated nature of the double bonds facilitates hydrogen abstraction, which initiates the lipid peroxidation cascade. Furthermore, a relatively high quantity of ROS was also physiologically produced by sperm mitochondria during the oxidative chain, which is necessary for providing enough energy to spermatozoa movement [11].

Based on what has been reported, it was not surprising that AgNPs affected the enzymes involved in oxidative protection (*Antioxidants*). In both the AgNP and LPS groups, the *Antioxidant* scores were lower than those of the control group, which was in agreement with other studies [47].

The endogenous free radical scavenging enzymes in semen include superoxide dismutase (SOD), CAT, and GPX [48]. Semen has a relatively low quantity of these enzymes [49, 50], and their reduction is probably associated with damage to sperm cell structure caused by nanoparticle administration. The oxidative damage improved strongly with NSAID administration, whereas Vit E only partially reverse the oxidative thrust, suggesting that the main negative effect of AgNPs was due to a proinflammatory response rather than to an oxidative response. Similarly, Yin et al. [30] demonstrated that vitamin E supplementation (100 mg/kg/day) attenuated neurotoxicity effects induced by nasal administration of AgNPs (2 mg/kg/day) in rats without completely mitigating them.

Although oxidative stress seemed to be of subordinate importance in our case, it is recognized as one of the major contributions to defective sperm. Indeed, mitochondrial DNA is particularly vulnerable to free radical attack because it is no hardly protected [51, 52]. In contrast, nucleic DNA is more protected than mitochondrial DNA; however, during inflammation, the leucocytes that are thought be a membrane barrier could activate oxygen or nitrogen species and generate free radicals, subsequently causing DNA damage [53]. In mice, decreased sperm chromatin condensation was reported when AgNPs were administered for 35 days, which was also associated with NOS and ROS production [43]. Accordingly, Fathi et al. [54] demonstrated that the adverse effect of AgNPs on sperm and seminiferous tubes of male rats was dose-dependent, showing worse trends with 300 mg/kg AgNP administration than with that of 30 mg/kg. Furthermore, the nature of particles also influenced their effects: Ag particles of submicron size (200 nm) caused genotoxicity (DNA fragmentation) in the testes and lungs of mice more severely than that induced by nanosized particles (20 nm) [55, 56].

## 5. Conclusion

Taking these results into consideration, it was possible to assume that exposure to AgNPs produced a similar but more persistent effect than that of LPS on rabbit reproductive tissues. AgNP administration triggered a proinflammatory response (increased *cytokines*), also linked to oxidative thrust (reduced *Antioxidant* and increased *Oxidative metabolites* scores), which, in turn, promoted AgNP crossing of the BTB, worsening many sperm outcomes (*Motility*). However, only anti-inflammatory treatment counteracted the negative effects of AgNPs, whereas vitamin E seemed to act only as an adjuvant, attenuating the oxidative cascade. Further studies to evaluate the synergistic effect of NSAID and Vit E administration on the sperm characteristics of NP-treated animals should be performed.

In conclusion, the present results confirmed the negative effect of AgNPs on reproductive tissues, underlining a progressive effect: (i) synergic inflammatory/oxidative thrust that impairs the reproductive environment, affecting the physiological status of sperm, and (ii) DNA damage due to AgNP crossing of the BTB with negative effects on the generation of new sperm waves. Furthermore, we demonstrated that such problems could be counteracted with anti-inflammatory administration rather than that of antioxidants.

## Figures and Tables

**Table 1:** Loadings of the principal components (PCs) extracted from principal component analysis (PCA).

PCA and items	PC		
PCA1	Motility		Volume
Sperm motility	*0.817*		-0.108
VCL	*0.795*		0.241
Semen volume	0.159		*0.863*
Sperm concentration	0.498		*-0.524*
PCA2	Antioxidants	Oxidative metabolites	Redox reaction
GPX blood	*0.847*	0.142	-0.081
GPX sperm	*0.846*	-0.109	-0.069
NO blood	0.306	*0.740*	0.082
MDA sperm	-0.044	*0.737*	0.084
MDA seminal plasma	-0.370	*0.637*	0.124
Catalase blood	0.057	0.088	*-0.901*
NO sperm	-0.056	0.306	*0.802*
MDA blood	-0.317	0.246	0.354
PCA3	TNF-*α* and IL-8		IL-6
TNF-*α*	*0.839*		0.183
IL-8	*0.830*		-0.338
IL-1*β*	*0.652*		0.470
IL-6	0.008		*0.925*

Loadings of >|0.5| are bolded. PCA: principal component analysis; PC: principal component; VCL: curvilinear velocity; GPX: glutathione peroxidase; SP: seminal plasma; TNF-*α*: tumor necrosis factor alpha; IL: interleukin.

**Table 2:** Estimated marginal means and standard error of principal component (PC) scores in rabbits treated with a saline solution (control group), AgNPs (AgNP group), or lipopolysaccharides (LPS group).

PC	Group^1^			*P* value		
	Control	AgNPs	LPS			
				Group	Time	Interaction
Motility	0.616_a_ ± 0.011	0.273_c_ ± 0.022	0.356_b_ ± 0.021	<0.001	<0.001	<0.001
Volume	0.380_a_ ± 0.011	0.331_a_ ± 0.007	0.342_a_ ± 0.010	0.443	0.012	0.006
Antioxidants	0.563_a_ ± .023	0.324_b_ ± 0.022	0.354_b_ ± 0.028	<0.001	<0.001	<0.001
Oxidative metabolites	0.286_c_ ± 0.014	0.522_a_ ± 0.032	0.419_b_ ± 0.030	<0.001	<0.001	<0.001
Redox reaction	0.343_b_ ± 0.013	0.537_a_ ± 0.028	0.504_a_ ± 0.023	<0.001	<0.001	<0.001
TNF-*α* and IL-8	0.231_b_ ± 0.008	0.357_a_ ± 0.025	0.375_a_ ± 0.032	<0.001	<0.001	<0.001
IL-6	0.223_b_ ± 0.009	0.394_a_ ± 0.028	0.383_a_ ± 0.031	<0.001	<0.001	<0.001

Values in the same row not sharing the same subscript are significantly different at *P* < 0.05 (Sidak correction). PC: principal component; TNF-*α*: tumor necrosis factor alpha; IL: interleukin. ^1^Control group, treated with 2 ml of 0.9% saline solution; AgNP group, treated with 5 mM AgNP solution; LPS group, inoculated with 50 g/kg b.w. of *E. coli* LPS.

**Table 3:** Estimated marginal means and standard error of principal component (PC) scores in rabbits treated with AgNPs (AgNP group), with AgNPs and an anti-inflammatory (AgNPs + NSAID group) or with AgNPs and vitamin E (AgNPs + Vit E group).

PC	Group^1^			*P* value		
	AgNPs	AgNPs + NSAID	AgNPs + Vit E			
				Group	Time	Interaction
Motility	0.273_c_ ± 0.022	0.470_a_ ± 0.013	0.402_b_ ± 0.018	<0.001	<0.001	<0.001
Volume	0.331_b_ ± 0.007	0.468_a_ ± 0.011	0.390_b_ ± 0.009	<0.001	0.401	<0.001
Antioxidants	0.324_b_ ± 0.022	0.543_a_ ± 0.025	0.346_b_ ± 0.014	<0.001	<0.001	<0.001
Oxidative metabolites	0.522_a_ ± 0.032	0.525_a_ ± 0.032	0.543_a_ ± 0.024	0.548	<0.001	<0.001
Redox reaction	0.537_a_ ± 0.028	0.388_c_ ± 0.017	0.484_b_ ± 0.023	<0.001	<0.001	<0.001
TNF-*α* and IL-8	0.357_a_ ± 0.025	0.270_b_ ± 0.006	0.357_a_ ± 0.031	<0.001	<0.001	<0.001
IL-6	0.394_a_ ± 0.028	0.286_b_ ± 0.016	0.443_a_ ± 0.028	<0.001	<0.001	<0.001

Values in the same row not sharing the same subscript are significantly different at *P* < 0.05 (Sidak correction).^1^AgNP group, treated with 5 mM AgNP solution; AgNPs + NSAID group, treated with 0.2 mg/kg b.w of anti-inflammatory and 5 mM AgNP solution. AgNPs + Vit E group, treated with 0.18 mg/kg b.w. of *α*-tocopheryl acetate and 5 mM AgNP solution.

## Data Availability

All data and figures used to support the findings of this study are included within the article.

## Abbreviations

AgNPs: Silver nanoparticles
BTB: Blood-testis barrier
TEM: Transmission electron microscopy
ROS: Reactive oxygen species
NOS: Reactive nitrogen species
LPS: Bacterial lipopolysaccharide
NSAIDs: Nonsteroidal anti-inflammatory drugs
PVP: Polyvinylpyrrolidone
EDTA: Ethylenediaminetetraacetic acid
SP: Seminal plasma
VCL: Curvilinear velocity
MDA: Malondialdehyde
CAT: Catalase
NO: Nitric oxide
GPX: Glutathione peroxidase
PBS: Phosphate-buffered saline
BHT: Butylated hydroxytoluene
NADPH: Nicotinamide adenine dinucleotide phosphate
TNF: Tumor necrosis factor
IL: Interleukin
LMM: Linear mixed model
PCA: Principal component analysis
PC: Principal components
ROMs: Reactive oxygen metabolites
DNA: Deoxyribonucleic acid.

## Supplementary Materials

Supplementary Materials Table S1: effects of LPS and AgNPs. Values are estimated marginal means and standard error of mean. Table S2: effects of NSAID and Vit E on AgNP-induced inflammation. Values are estimated marginal means and standard error of mean.
