# Supplementary material for: Oxidative and/or Inflammatory Thrust Induced by Silver Nanoparticles in Rabbits: Effect of Vitamin E or NSAID Administration on Semen Parameters
Source: Mediators Inflamm. 2020 Dec 26;2020:6664062. doi: 10.1155/2020/6664062 (PMC7781726; doi:10.1155/2020/6664062)
Supplement: Supplementary Materials — Table S1: effects of LPS and AgNPs. Values are estimated marginal means and standard error of mean. Table S2: effects of NSAID and Vit E on AgNP-induced inflammation. Values are estimated marginal means and standard error of mean. [file 6664062.f1.docx]

**Supplementary materials**

**Table S1.** Effects of LPS and AgNPs. Values are Estimated marginal means and Standard Error of Mean

|  | **Group** | | | **P value** | | |
| --- | --- | --- | --- | --- | --- | --- |
|  | **Control** | **AgNPs** | **LPS** |  |  |  |
|  |  |  |  | **TRT** | **Time** | **Interaction** |
| **Volume (µL)** | 567.77_a_±14.99 | 471.58_a_±12.53 | 464.61_a_±18.81 | 0.083 | <0.001 | 0.006 |
| **Sperm concentration (10^6^/mL)** | 368.61_a_±9.92 | 292.95b±14.82 | 301.72_b_±19.05 | 0.028 | <0.001 | 0.003 |
| **Static (%)** | 0.24_b_±0.01 | 0.41_a_±0.02 | 0.38_a_±0.01 | <0.001 | <0.001 | <0.001 |
| **Motility (%)** | 0.76_a_±0.01 | 0.59_b_±0.02 | 0.62_b_±0.01 | <0.001 | <0.001 | <0.001 |
| **VCL (μm/s)** | 295.94_a_±3.41 | 184.68_c_±4.98 | 224.39_b_±4.91 | <0.001 | <0.001 | 0.005 |
| **MDA SP (nmol /mL)** | 0.89_c_±0.08 | 2.94_a_±0.37 | 2.12_b_±0.28 | <0.001 | <0.001 | <0.001 |
| **MDA sperm (pmol /10^8^ cells)** | 5.02_c_±0.30 | 9.85_a_±0.59 | 6.23_b_±0.37 | <0.001 | <0.001 | <0.001 |
| **MDA blood (nmol /mL)** | 0.47_b_±0.02 | 0.75_a_±0.06 | 0.78a±0.05 | <0.001 | <0.001 | <0.001 |
| **GPX blood (U/10^6^ cells)** | 155.80_a_±12.37 | 62.46_b_±5.40 | 68.09_b_±6.09 | <0.001 | <0.001 | <0.001 |
| **GPX sperm (U/10^6^ cells)** | 0.99a±0.04 | 0.44_b_±0.03 | 0.49_b_±0.05 | <0.001 | <0.001 | <0.001 |
| **CAT sperm (U/10^6^ cells)** | 115.19_a_±4.66 | 63.65_c_±6.27 | 77.08_b_±6.24 | <0.001 | <0.001 | <0.001 |
| **CAT blood (U/10^6^ cells)** | 225.36_a_±8.45 | 121.36_c_±10.80 | 149.62_b_±10.02 | <0.001 | <0.001 | <0.001 |
| **NO SP (µM)** | 66.10_a_±2.31 | 90.70_a_±3.96 | 94.05_b_±4.54 | <0.001 | <0.001 | <0.001 |
| **NO sperm (nmol/10^6^ cells)** | 2.63_c_±0.08 | 4.81_a_±0.29 | 4.23_b_±0.18 | <0.001 | <0.001 | <0.001 |
| **IL-6 (pg/mL)** | 0.31_b_±0.01 | 0.51_a_±0.05 | 0.50_a_±0.04 | <0.001 | <0.001 | <0.001 |
| **IL-8 (pg/ mL)** | 2.28_b_±0.02 | 2.79_a_±0.20 | 2.65_a_±0.16 | <0.001 | <0.001 | <0.001 |
| **TNF-α (pg/ mL)*** | 0.03_c_±0.00 | 0.05_b_±0.00 | 0.13_a_±0.00 | <0.001 | <0.001 | <0.001 |
| **IL-1β (pg/ mL)** | 27.34_c_±0.36 | 38.21_a_±0.84 | 34.09_b_±0.74 | <0.001 | <0.001 | <0.001 |

*back transformed values.

Values in the same row not sharing the same subscript are significantly different at P < 0.05 (Sidak correction).

**Table S2.** Effects of NSAID and vit E on AgNPs -induced inflammation. Values are Estimated marginal means and Standard Error of Mean

|  | **Group** | | | **P value** | | |
| --- | --- | --- | --- | --- | --- | --- |
|  | **AgNPs** | **AgNPs + NSAID** | **AgNPs + Vit E** |  |  |  |
|  |  |  |  | **TRT** | **Time** | **Interaction** |
| **Volume (µL)** | 472.14_b_±12.46 | 617.15_a_±27.84 | 499.76_b_±10.73 | 0.005 | 0.002 | <0.001 |
| **Sperm cell (10^6^/mL)** | 292.95_a_±14.82 | 224.15_b_±8.79 | 259.77_ab_±7.95 | 0.018 | 0.002 | 0.008 |
| **Static (%)** | 0.41_a_±0.02 | 0.32_c_±0.01 | 0.36_b_±0.01 | <0.001 | <0.001 | <0.001 |
| **Motility (%)** | 0.59_c_±0.02 | 0.68_a_±0.01 | 0.64_b_±0.01 | <0.001 | <0.001 | <0.001 |
| **VCL (μm/s)** | 184.68_c_±4.98 | 280.18_a_±7.34 | 253.24_b_±7.43 | <0.001 | <0.001 | <0.001 |
| **MDA SP (nmol /mL)** | 2.94_a_±0.37 | 1.46_b_±.17 | 2.92_a_±0.21 | <0.001 | <0.001 | <0.001 |
| **MDA sperm (pmol /10^8^ cells)** | 9.86_a_±0.59 | 9.43_a_±0.59 | 9.58_a_±0.47 | 0.722 | <0.001 | <0.001 |
| **MDA blood (nmol /mL)** | 0.75_a_±0.06 | 0.53_b_±0.01 | 0.68_a_±0.03 | <0.001 | <0.001 | <0.001 |
| **GPX blood (U/10^6^ cells)** | 62.44_c_±5.40 | 146.75_a_±14.52 | 82.55_b_±7.07 | <0.001 | <0.001 | <0.001 |
| **GPX sperm (U/10^6^ cells)** | 0.44_b_±0.03 | 0.87_a_±0.04 | 0.48_b_±0.02 | <0.001 | <0.001 | <0.001 |
| **CAT sperm (U/10^6^ cells)** | 63.65_c_±6.27 | 100.06_a_±5.58 | 82.93_b_±4.11 | <0.001 | <0.001 | <0.001 |
| **CAT blood (U/10^6^ cells)** | 121.03_c_±10.82 | 199.88_a_±11.22 | 165.95_b_±8.22 | <0.001 | <0.001 | <0.001 |
| **NO SP (µM)** | 90.69_b_±3.96 | 103.95_a_±3.74 | 99.31_a_±3.38 | <0.001 | <0.001 | <0.001 |
| **NO sperm (nmol 10^6^ cells)** | 4.81_a_±0.29 | 3.52_b_±0.25 | 4.50_a_±0.25 | <0.001 | <0.001 | <0.001 |
| **IL-6 (pg/mL)** | 0.51_b_±0.05 | 0.43_b_±0.02 | 0.65_a_±0.05 | <0.001 | <0.001 | <0.001 |
| **IL-8 (pg/ mL)** | 2.79_a_±0.20 | 2.72_a_±0.04 | 2.70_a_±0.14 | 0.803 | <0.001 | <0.001 |
| **TNF-α (pg/ mL)*** | 0.05_b_±0.00 | 0.02_a_±0.00 | 0.07_b_±0.00 | <0.001 | <0.001 | <0.001 |
| **IL-1β (pg/ mL)** | 38.21_a_±0.84 | 28.92_c_±0.95 | 35.18_b_±0.85 | <0.001 | <0.001 | <0.001 |

*back transformed values.

Values in the same row not sharing the same subscript are significantly different at P < 0.05 (Sidak correction).
